# The End-Results of Cases Operated on for Gallstones
^1^Read at a meeting of the Bath and Bristol Branch of the British Medical Association, held at Bristol on October 30th, 1912.


**Published:** 1913-03

**Authors:** A. Rendle Short

**Affiliations:** Assistant Surgeon, Bristol Royal Infirmary; Demonstrator of Physiology, University of Bristol.


### THE END-RESULTS OF CASES OPERATED ON FOR GALLSTONES.[1]

BY

A. Rendle Short, M.D., B.S., B.Sc.Lond., F.R.C.S.,

*Assistant Surgeon, Bristol Royal Infirmary ;*
*Demonstrator of Physiology, University of Bristol.*

It is easy to obtain details as to the findings at operation, the mortality rate, and the condition of the patient a few weeks after any surgical procedure, but reliable figures collected by a disinterested observer, extending over a period of years afterwards, are few and far between, and it would be difficult, if not impossible, to obtain such guidance with reference to the operation for gallstones.

The end-results presented in this paper relate to patients operated on at the Bristol Royal Infirmary from January, 1900, to July, 1911. In most cases the details have been obtained by correspondence ; a small number have been examined

[1] Read at a meeting of the Bath and Bristol Branch of the British Medical Association, held at Bristol on October 30th, 1912.

personally. In every case the operation was performed at least fourteen months ago, and the great majority of the patients have been traced for several years.

It is remarkable how surgical work in this field has increased of late. In three years, 1900–2, seven cases were operated on for gallstones. In three years, 1908–10, the number had grown to forty-three.

Patients in whom a malignant growth was found as well as gallstones have been excluded from the tables.

It has not been the custom to operate at the Bristol Royal Infirmary unless there have been a number of attacks sufficient to lay the patient up frequently, or unless there is a palpable tumour or persistent jaundice. The practice of nine different surgeons is represented.

GENERAL TABLE OF RESULTS.

| | Cases. | Died in Hospital | Lost sight of. | Traced. | Cured. | Much better. | Little or no better. | Died since. |
|---|---|---|---|---|---|---|---|---|
| I. Stones in gall-bladder only. | 42 | 7 | 7 | 28 | 10 | 12 | 4 | 2 |
| II. Stones in common duct also. | 25 | 3 | 4 | 18 | 12 | 4 | 2 | 0 |
| III. Stones in cystic duct. | 12 | 0 | 2 | 10 | 6 | 2 | 2 | 0 |
| IV. Stones in hepatic duct. | 2 | 0 | 0 | 2 | 1 | 0 | 1 | 0 |
| V. No stones; adhesions, etc. | 2 | 1 | 1 | 0 | 0 | 0 | 0 | 0 |
| VI. Peritonitis present. | 2 | 0 | 0 | 2 | 1 | 1 | 0 | 0 |
| VII. Intestinal obstruction. | 1 | 1 | 0 | 0 | 0 | 0 | 0 | 0 |
| Total .. | 86 | 12 | 14 | 60 | 30 | 19 | 9 | 2 |
| Fever Cases | 9 | 2 | 0 | 7 | 4 | 1 | 1 | 1 |

*Age and Sex.*—The findings accord with the general description. Of 86 cases 13 were males and 73 females.

The age table is as follows: Under 20, 1 case; 20 to 30, 3 cases; 30 to 40, 18 cases; 40 to 50, 22 cases; 50 to 60, 27 cases; over 60, 16 cases.

The youngest case was 17, and the oldest 70.

*Clinical History and Operation Findings.*—It is not proposed to discuss here the symptoms of the various patients. The great majority had suffered from repeated attacks of colic, with jaundice. As a general rule, to which there were exceptions, when the patient was operated on in the presence of jaundice there were stones in the common duct. In one case, however, although jaundice and colic had persisted for two months, no stones were found, but the gall-bladder was shrivelled. This was in 1901; probably nowadays chronic pancreatitis might be recognised in such a case.

A girl aged 27 vomited a gallstone; apart from this the diagnosis would have been gastric ulcer or movable kidney. The gall-bladder was full of stones at the time of operation, but the ducts were not dilated, and no fistulæ were present.

In nine cases there were acute febrile symptoms with pain, tenderness, and "guarding" in the right hypochondrium. In two of these acute general peritonitis was present. They were diagnosed as acute abdominal catastrophes, probably gastric in origin. In one the gall-bladder was found perforated, with a stone inside it; in the other it was large, inflamed, and contained three stones, and no other cause for the peritonitis being found, it was drained. Both patients recovered well.

A woman aged 65 was admitted suffering from intestinal obstruction of fourteen days' duration, with stercoraceous vomiting. On opening the abdomen a stone was found in the small intestine ($3\frac{7}{8}$ by $4\frac{3}{8}$ by $1\frac{3}{8}$ inches). As usual in these cases, there was no history of gallstone colic. She died shortly afterwards.

It is noteworthy that quite a number of the patients stated that they had suffered from catarrhal jaundice in their teens. Three had had enteric fever.

*Operation Mortality.*—Out of 86 cases 12 died in the Infirmary, but it is not fair to take this as the mortality of ordinary uncomplicated gallstone operations. Deducting the cases in which febrile symptoms and signs of local sepsis were present, and also the fatal case of intestinal obstruction, we are left with nine deaths in 76 cases (about 12 per cent.). This figure is undoubtedly higher than the true average. One patient died suddenly twenty-one days after the operation, and at the autopsy nothing could be found but a fatty heart. Two cases died of gastric ulcer which had not been diagnosed previously. The first, a man of 60, succumbed to profuse hæmatemesis on the sixteenth day, and at the autopsy an ulcer was found opening an artery. In this patient the portal vein had been punctured at the operation, and the hole closed, but the vein appears to have been normal at the *post-mortem*. The other case, a man of 31, died of perforated gastric ulcer on the ninth day.

In another patient, a woman, the portal vein was opened and bled furiously, so that nothing could be done but apply large forceps and tie off in the dark; she died a few hours later, and at the autopsy the vein was found to be pulled by the abundant adhesions right in front of the bile-duct, so that it would have been very difficult to avoid the catastrophe.

In most published figures (*e.g.* the Mayos') the mortality of common duct cases is much higner than that for stones in the gall-bladder only, but this is not so in our figures, the preponderance being, strange to say, in the other direction. Our mortality (two out of nine) in febrile cases is, naturally, above the average for afebrile cases.

Adhesions complicate the operation seriously. Out of nineteen cases in which abundant adhesions are mentioned in the notes, six died in the institution (about 30 per cent.).

The causes of death were as follows: Shock, 3 cases; sepsis or peritonitis, 3 cases; gastric ulcer, perforated, 1 case (nine days after); gastric ulcer, bleeding, 1 case (sixteen days after); fatty heart, 1 case (twenty-one days after); pneumonia, 1 case; cut portal vein, 1 case; intestinal obstruction, 1 case.

*The End-results*.—Of seventy-four surviving patients, fourteen have been lost sight of, and sixty traced from fourteen months up to ten years after the operation.

Taking these all together, thirty describe themselves as perfectly cured, with no symptoms at all relating to gall-stones. When one remembers that this refers to middle-aged and usually corpulent working-class women, it will be admitted that it is a remarkably good result. Another nineteen patients are described as much better, meaning that there has been slight pain occasionally, but on the whole very great improvement; three classed here have had no further gallstone trouble, but suffer from incisional hernia. None of the cases in this group have asked for further treatment. Thus it will be seen that forty-nine out of sixty patients are very greatly relieved by the operation (82 per cent.).

In 9 out of 59 cases there has been but little relief. In one of these a stone was lost and knowingly left in the hepatic duct. Four cases had a second operation. In three of these nothing was found but adhesions; in the fourth there was a stone in the gall-bladder, but as there were a great number present at the original operation it is quite possible that this was not a new formation. Except in this doubtful case, the drainage of the gall-bladder would seem to have cured the catarrh which allows fresh stones to form. Recurrence of the pain is probably due in most instances to adhesions. Second operation did not bring much relief in any of these four patients, although the adhesions were divided.

Two patients have died since leaving. One died a month afterwards, but details are lacking. The other was quite cured of her gallstone trouble, but was operated on a year later for sarcoma of the pelvis, which proved to be irremovable.

There is not much difference in the end-results according to whether the stone is in the gall-bladder, common duct or cystic duct, except that adhesions are commoner with the first of these, and therefore absolute cures are less frequent. The extent of the adhesions is the principal factor in determining both the immediate and the ultimate result. It has been mentioned that out of 19 cases with extensive adhesions 6 died; of 11 whose after-history is known 3 are quite cured, 4 much improved, and 4 little or no better. The failures in the absence of adhesions are only 10 per cent.; when they are present the failures amount to 36 per cent.

The usual operation has been to remove the stones and to drain the gall-bladder, and common duct as well in some cases. If the gall-bladder was extensively diseased it was removed. The results in these ten patients, treated by cholecystectomy, were not so good, but the figures are small, and relate to the severer type of cases. Three died, three are lost sight of, and of four followed throughout three are practically cured; but one, operated on in 1910, has had two or three severe attacks of pain, with jaundice since. It would appear as if the stones have reformed in the hepatic ducts.

In a patient in whom the gall-bladder was found distended with turbid fluid and drained in 1902, there has been no return of symptoms, but since 1910 she has been suffering from diabetes, possibly due to an ascending infection of the pancreatic as well as of the bile-duct.

In two or three cases there was a single attack of gallstone colic a week or two after the operation, but none whatever since, even over a long period of years. Possibly one small stone was overlooked, or there may have been an attempt to expel blood-clot.

Although it is well known that cancer may supervene upon gallstones, several instances having occurred in the Infirmary of both being found together at the operation, it is perhaps worthy of note that in none of our 60 cases, followed for more than a year, has cancer developed so far. The argument for cholecystectomy to avoid malignant disease is therefore not very strong.

### Summary.

1. Of 86 patients operated on for gallstones 12 died, but the mortality in ordinary and febrile cases was 12 per cent. Unlike most statistics, the mortality was higher in removals of the stone from the gall-bladder than from the common duct.

2. Of 60 patients followed through for more than a year, about 82 per cent. are quite cured, or very greatly improved. Failure is usually due to adhesions, not to recurrence of the stone formation.

3. Where adhesions were found to be extensive, 6 out of 19 died. Of those traced, 4 out of 11 were but little improved (36 per cent. failures). Apart from adhesions, there were only 10 per cent. failures.

4. According to our figures, of 100 ordinary afebrile cases operated on, 12 died, 75 are greatly improved or quite cured, and 13 are not much bettered.

5. Cases are related of intestinal obstruction by a gallstone, vomiting a stone, injury of the portal vein, general peritonitis due to gallstones, deaths from perforating gastric ulcer, and from hæmatemesis caused by a gastric ulcer soon after operation for gallstones, and diabetes supervening long afterwards.

